# Multiple Minor QTLs Are Responsible for Fusarium Head Blight Resistance in Chinese Wheat Landrace Haiyanzhong

**DOI:** 10.1371/journal.pone.0163292

**Published:** 2016-09-27

**Authors:** Jin Cai, Shan Wang, Tao Li, Guorong Zhang, Guihua Bai

**Affiliations:** 1 Department of Agronomy, Kansas State University, Manhattan, Kansas, United States of America; 2 Institute of Food Crops, Jiangsu Academy of Agricultural Sciences, Nanjing, Jiangsu, China; 3 Department of Agronomy, Yangzhou University, Yangzhou, Jiangsu, China; 4 USDA Hard Winter Wheat Genetics Research Unit, Manhattan, Kansas, United States of America; Huazhong University of Science and Technology, CHINA

## Abstract

*Fusarium* head blight (FHB), caused by *Fusarium graminearum* Schwabe, is a devastating disease in wheat (*Triticum aestivum* L.). Use of host resistance is one of the most effective strategies to minimize the disease damage. Haiyanzhong (HYZ) is a Chinese wheat landrace that shows a high level of resistance to FHB spread within a spike (type II resistance). To map the quantitative trait loci (QTLs) in HYZ and identify markers tightly linked to the QTLs for FHB resistance, a population of 172 recombinant inbred lines (RILs) from a cross between HYZ and Wheaton (FHB susceptible) was genotyped using simple sequence repeats (SSRs) and single-nucleotide polymorphisms (SNPs) derived from genotyping-by-sequencing (GBS), and evaluated for percentage of symptomatic spikelets (PSSs) per spike in three greenhouse experiments. Six QTLs for type II resistance were identified in HYZ, indicating that multiple minor QTLs together can provide a high level of FHB resistance in wheat. The QTL with the largest effect on FHB resistance was mapped on the chromosome arm 5AS, and the other five from HYZ were mapped on the chromosomes 6B, 7D, 3B, 4B and 4D. In addition, two QTLs from Wheaton were mapped on 2B. Critical SNPs linked to the QTLs on chromosomes 5A, 6B, and 2B were converted into KBioscience competitive allele-specific PCR (KASP) assays, which can be used for marker-assisted selection (MAS) to pyramid these QTLs in wheat.

## Introduction

*Fusarium* head blight (FHB), mainly caused by *Fusarium graminearum* Schwabe, is one of the most destructive diseases of wheat (*Triticum aestivum*), especially in humid and semi-humid wheat-growing regions of the world [1, 2]. It causes significant reduction in grain yield and quality. Infected grain is also contaminated with mycotoxins, especially deoxynivalenol (DON), which is a major health concern for humans and animals [3]. Although progress has been made in managing FHB during the last several decades, economic losses from FHB and DON are growing in many regions in the U.S. and many other countries due to change in climate and cropping system [4]. No single strategy is completely effective in alleviating FHB damage. However, growing FHB-resistant cultivars coupled with appropriate cultural practices can minimize FHB damage.

FHB resistance in wheat is a quantitative trait controlled by multiple quantitative trait loci (QTLs) and is affected by environmental factors [5, 6]. To date, more than 50 QTLs for FHB resistance have been reported on all 21 chromosomes [7, 8]. Seven QTLs have been formally designated with gene names from *Fhb1* to *Fhb7* [9–15]. However, most of the QTLs were mapped using low-density maps, and high density maps are critical to the identification of tightly linked markers to these QTLs. Genotyping-by-sequencing (GBS) is a simple, but effective, approach for simultaneous discovery and mapping of SNP markers in diverse species [16], and is a useful marker system for fine mapping of QTLs for FHB resistance.

FHB resistance genes used in most wheat breeding programs can be traced back to very few sources with most of them derived from Sumai3 [1]. Limited sources of resistance used in breeding could create vulnerability to resistance breakdown by evolving pathogen populations. Exploring new sources of resistance will facilitate pyramiding of different QTLs to increase the resistance level and diversity of resistant sources. Several Chinese landraces showed as high level of FHB resistance as Sumai3, including Haiyanzhong (HYZ) [17]. Li et al. (2011) did not find *Fhb1*, the most common QTL for FHB resistance in Chinese sources, in HYZ using a population of 136 recombinant inbred lines (RILs) of HYZ x Wheaton [18]. Instead, they identified a major QTL on 7DL, and suggested that HYZ might be a different source of resistance from Sumai3. The objectives of the present study were to (1) validate the previous mapped QTL on 7D in HYZ using a new larger population; (2) identify possible new QTLs using a high density SNP map; and (3) develop tightly linked markers for marker-assisted selection (MAS).

## Materials and Methods

### Plant materials and FHB evaluation

A population of 186 F_7_-derived RILs was developed from a cross between HYZ and a U.S. FHB-susceptible hard red spring wheat variety, Wheaton, by single-seed descent. The RILs were evaluated for FHB resistance in the greenhouses in spring and fall 2012, and spring 2013 at Kansas State University in Manhattan, Kansas. Seeds of the RILs and two parents were planted in plastic trays filled with Metro-mix 360 soil mix (Hummert International, Topeka, KS). After 50 d of vernalization at 6°C in a cold room, about six seedlings per line were transplanted into a 14 x 14 cm Dura pot filled with Metro-mix 360 soil mix. The pots were arranged on greenhouse benches in a randomized complete block design (RCBD) with two replications (pots) per line. The greenhouse was maintained at 17 ± 2°C at night and 22 ± 5°C during a day with 12 h supplemental daylight. An association mapping (AM) population of 96 U.S. elite wheat accessions was used to check the marker allele distribution in US winter wheat (S1 Table).

A Kansas strain of *F*. *graminearum* (GZ3639) was used as an inoculum, and a conidial spore suspension was prepared following Bai et al. (1999) [19]. At early anthesis, wheat spikes were inoculated by injecting 10 μl of a conidial spore suspension (~1000 spores/spike) into a floret of a central spikelet in a spike using a syringe (Hamilton, Reno, NV). Five spikes per pot were inoculated and maintained in a moist chamber at 100% relative humidity and 20 to 22°C for 48 h to initiate fungal infection. Then the plants were returned to the greenhouse benches for further FHB development. FHB symptom spread within a spike (type II resistance) was evaluated by counting the symptomatic spikelets and total spikelets in each inoculated spike 15 d after inoculation. Percentage of symptomatic spikelets (PSS) in a spike from each RIL in each experiment and mean PSS across all three experiments were calculated for QTL analysis.

### DNA extraction and marker analysis

Leaf tissue was collected at the three-leaf stage in 96-deepwell plates, dried in a freeze dryer (ThermoSavant, Holbrook, NY) for 48 h, and ground using a Mixer Mill (MM 400, Retsch, Germany). Genomic DNA was isolated using a modified cetyltrimethyl ammonium bromide protocol [20].

A core set of 384 simple sequence repeat (SSR) primers that are highly polymorphic and cover all the 21 wheat chromosomes [21] were used to screen the two parents. This primer set was originally selected from 2000 primer pairs (http://wheat.pw.usda.gov) based on the result from previous studies conducted at the USDA Central Small Grain Genotyping Laboratory in Manhattan, KS. Primers that amplified at least one polymorphic band between the parents were used to screen the 186 RILs. Polymerase chain reaction (PCR) amplification and SSR detection followed [22]. Data were scored using GeneMarker v1.75 (SoftGenetics LLC, State Collage, PA).

A GBS library was generated from RILs and parents using a previously described protocol [16]. In brief, each DNA sample was digested with *HF-PstI* (High-Fidelity) and *MspI* and ligated with adaptors using T4 ligase (New England BioLabs Inc., Ipswich, MA). Ligated samples with different barcodes were pooled into a single tube, cleaned up using a QIAquick PCR Purification Kit (Qiagen Inc., Valencia, CA), and then amplified by PCR using 10 μM Ion primers and 5 μl *Taq* 5X Master Mix (New England BioLabs Inc.). The PCR mixture was incubated at 95°C for 30 sec initially, followed by 16 cycles of 95°C for 30 sec, 62°C for 20 sec, and 68°C for 1min, then at 72°C for 5 min for a final extension. The PCR products were cleaned up again using the QIAquick PCR Purification Kit, and selected 250–300 bp fragments in an E-gel system (Life Technologies Inc.) for sequencing in an Ion Proton system (Life Technologies Inc.). GBS data generated from Ion Proton were analyzed for SNPs using UNEAK, an independent reference pipeline of TASSEL [16, 23]. For these sequence reads with less than 64 bp, a poly-A tail was added to the reads to ensure all reads were 64 bp.

The accuracy of GBS-SNP calls was validated using KBioscience allele-specific PCR (KASP) assays (LGC Genomics, Beverly, MA). The KASP primers were designed from the corresponding GBS sequences harboring the SNPs that were mapped to the QTL regions. The KASP master mix for each reaction comprised of 3 μl of 2x KASP reaction mix, 0.0825 μl of KASP primer mix (100 μM) and 3 μl of DNA (~40 ng). Samples were incubated at 94°C for 15 min, followed by 10 cycles of 94°C for 20 s and annealing at 65°C for 1 min with a decrease of 0.8°C in each subsequent cycle. Then the PCR went through an additional 40 cycles of 94°C for 20 sec and 57°C for 1 min. After PCR, plates were read in an Applied Biosystems 7900HT Fast Real-Time PCR System (Life Technologies Inc.). The mismatches between GBS-SNP and KASP-SNP data were counted. If any mismatch, the KASP markers were remapped together with other GBS-SNPs to determine their map locations.

### Genetic map construction and QTL analysis

A linkage map with both SSR and GBS-SNP markers was constructed using the Kosambi mapping function [24] and ‘regression’ mapping algorithm in JoinMap version 4.0 [25]. QTLs for PSS were determined using Composite Interval Mapping (CIM) in WINQTL Cartographer version 2.5 with Model 6 [26]. The permutation test was performed 1000 times to determine the LOD threshold for claiming significant QTLs at *P* < 0.05 [27].

## Results

### FHB disease severity variation among RILs and between parents

**Fig 1:**
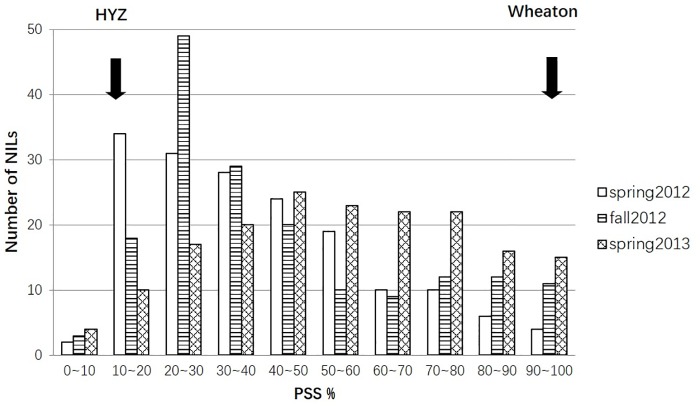
Frequency distribution of mean percentage of symptomatic spikelets in a spike (PSS) for the recombinant inbred line (RIL) population derived from ‘Haiyanzhong’ (HYZ) x ‘Wheaton’ evaluated in spring and fall 2012, and spring 2013 greenhouse experiments.

The resistant parent HYZ showed a high level of FHB resistance in all three greenhouse experiments, with an average PSS of 11.2%, ranging from 7.6 to 14.8%, whereas the susceptible parent Wheaton had a mean PSS of 97.8%, ranging from 95.5 to 100% (Fig 1), indicating a large contrast in PSS between the two parents. The mean PSSs of RILs across all the three experiments ranged from 7.6% to 100%. PSS frequencies showed continuous distribution skewed toward HYZ in spring and fall 2012, but toward Wheaton in spring 2013 (Fig 1). Mean PSS over all RILs was 46.2%, ranging from 39.9% (spring 2012) to 55.3% (spring 2013), indicating the highest disease pressure in spring 2013 and the lowest in spring 2012. Transgressive segregation was not evident in spring 2012, but obvious on fall 2012 and spring 2013, suggesting there might be QTL(s) contributed by the susceptible parent. The correlations were highly significant among the three greenhouse experiments, ranging from 0.58 to 0.64 (*P* < 0.001). Variations in genotypes, environments, and genotypes by environments were significant among the three experiments. The heritability was high (0.81).

### Construction of a linkage map

The GBS- SNPs were analyzed for 172 RILs after removing 14 RILs that had excessive missing data. After four Ion Proton runs, 21,740 SNPs were identified with ≤80% missing data. Among them, 6,232 SNPs had ≤20% missing data and were used for mapping. For SSR, 132 of 384 primer sets were polymorphic and used to screen all the RILs. Of the 6,364 markers (6,232 SNPs and 132 SSRs) analyzed in the mapping population, 4,624 (72.7%) were mapped to 48 linkage groups with at least three markers in each group. The map covered all 21 chromosomes at a genetic distance of 4,044.34 cM with an average marker density of 0.87 cM per maker. Among the three genomes of wheat, the B genome has the most markers (49.2%), followed by the A (40.8%) and D (10.0%) genomes. Marker density was the highest (0.50 cM per marker) in chromosome 3A, while the lowest (5.84 cM per marker) on chromosome 3D.

### QTLs for FHB resistance

**Fig 2:**
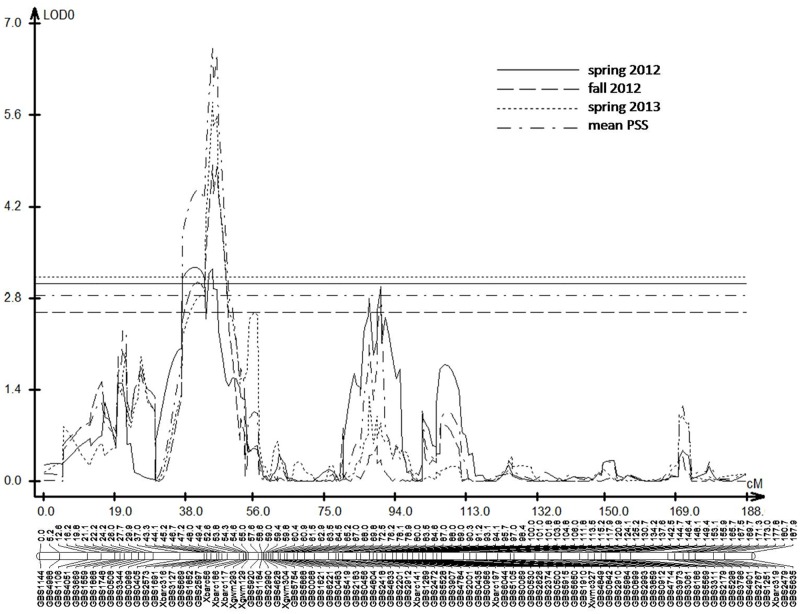
Maps of QTLs on 5A for FHB type II resistance constructed from the RIL population derived from the cross ‘HYZ’ x ‘Wheaton’ based on three greenhouse experiments.

**Fig 3:**
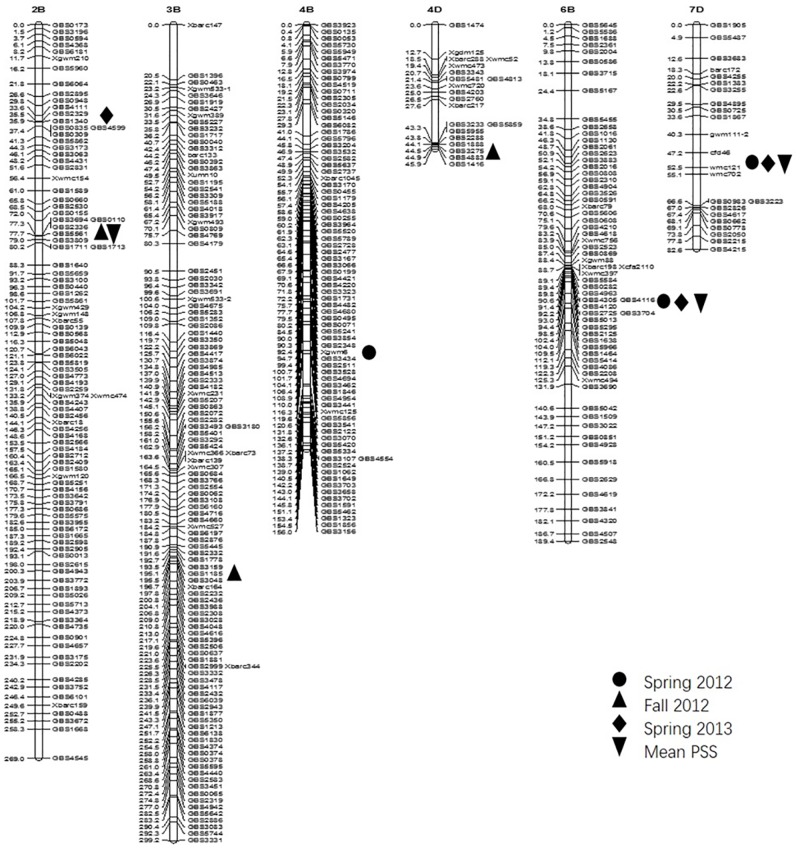
Maps of QTLs on 2B, 3B, 4B, 4D, 6B, and 7D for FHB type II resistance constructed from the RIL population derived from the cross ‘HYZ’ x ‘Wheaton’.

CIM mapping detected eight significant QTLs for FHB resistance on 5AS, 6BS, 7DL, 2B (two QTLs), 4D, 3B and 4B (Figs 2 and 3). The QTLs on chromosome 5AS, 6BS and 7DL were significant in at least two experiments and were mapped in the same positions as previously reported [18], whereas the other five QTLs were significant in only one experiment and they are newly mapped QTLs in the current study. The 5A QTL showed the largest effect in all three experiments among all QTLs mapped and explained 6.1~16.0% of the phenotypic variation (Table 1, Fig 2). This QTL was delineated to a 1.9 cM interval between SNPs *GBS3127* and *Xbarc316* with the QTL peak at *GBS3127* (Table 2). The QTL on 6BS, flanked by SNPs *GBS4963* and *GBS3704*, was significant in spring 2012 and 2013 data, and in mean PSS data. This QTL explained 6.9~11.1% of the phenotypic variation (Table 1, Fig 3). Six SNPs were mapped within a 2.4 cM interval, with *GBS4305* and *GBS4116* showing the largest effect among them. The QTL on 7DL was flanked by *Xcfd46* and *Xwmc702* with the QTL peak at *Xcfd46*. Polymorphic SNPs were not mapped in the QTL region. The QTL was significant in spring 2012 and 2013, and for the mean PSS, which explained 5.6~7.5% of the phenotypic variation (Table 1, Fig 3).

**Table 1:** Flanking markers, logarithm of odds (LOD) values, coefficients of determination (R^2^) of the significant QTLs detected by inclusive composite interval mapping using the FHB severity data collected from spring and fall 2012, and spring 2013 greenhouse.

QTL location	Source^§^	Flanking markers	Spring 2012		Fall 2012		Spring 2013		Combined mean	
			LOD	R^2^%	LOD	R^2^%	LOD	R^2^%	LOD	R^2^%
**5AS**	HYZ	*GBS3127~Xbarc316*	3.31	6.10	4.85	10.26	5.82	12.15	6.63	15.98
**6BS**	HYZ	*GBS4963~GBS3704*	3.97	8.26	-	-	5.80	11.11	3.57	6.91
**7DL**	HYZ	*Xcfd46~Xwmc702*	3.47	6.32	-	-	3.59	7.53	2.86	5.59
**2B-1**	Wheaton	*GBS1340~GBS0835*	-	-	-	-	3.28	5.80	-	-
**2B-2**	Wheaton	*GBS5561~GBS0848*	-	-	2.81	5.10	-	-	3.67	7.77
**4D**	HYZ	*GBS3223~GBS4883*	-	-	6.06	14.54	-	-	-	-
**3B**	HYZ	*GBS1778~GBS3048*	-	-	3.83	8.21	-	-	-	-
**4B**	HYZ	*GBS2348~GBS3434*	3.03	5.61	-	-	-	-	-	-

Note: ‘-’ represents insignificant at *P* = 0.05.

**^§^** Parent that contributes the resistance allele of a QTL.

**Table 2:** Difference in FHB severity between FHB resistance (R) and susceptibility (S) alleles at the QTL on chromosome 5A as reflected by two closely linked markers, and coefficients of determination of the QTL estimated from HYZ x Wheaton RILs tested in spring and fall 2012 and spring 2013 greenhouse experiments.

Marker	Allele	Spring 2012	Fall 2012	Spring 2013	Mean PSS
***GBS3127***	HYZ	34.31	37.18	48.33	39.99
	Wheaton	47.18	51.81	63.96	54.23
	Diff.	12.87*	14.63*	15.63*	14.24*
	R^2^	0.0867	0.0844	0.1090	0.1242
***Xbarc316***	HYZ	34.23	35.73	46.31	38.73
	Wheaton	44.45	49.68	62.65	52.22
	Diff.	10.22*	13.95*	16.35*	13.49*
	R^2^	0.0559	0.0794	0.1143	0.1120

Note:

* refers significant difference in PSSs between ‘HYZ’ and ‘Wheaton’ groups.

Five other minor QTLs were each detected in only a single experiment. Two QTLs for FHB resistance were mapped on the short arm of chromosome 2B. The susceptible parent Wheaton contributed positive alleles for those QTLs. The QTL 2B-1, flanked by SNPs *GBS1340* and *GBS0835*, was significant in spring 2013 only and explained 5.8% of the phenotypic variation (Table 1, Fig 3), whereas the QTL 2B-2 in a 3.3 cM interval between SNPs *GBS5561* and *GBS0848* was 40 cM away from the QTL 2B-1. This QTL was significant on fall 2012 and mean PSS, and explained 5.1~7.8% of the phenotypic variation (Table 1, Fig 3). The third minor QTL on chromosome 4DS was mapped between SNPs *GBS3233* and *GBS4883* and significant on fall 2012 only, which explained 14.5% of the phenotypic variation (Table 1, Fig 3). The fourth QTL on the long arm of chromosome 3B that flanked by SNPs *GBS1778* and *GBS3048* was significant in the fall 2012 experiment, and explained 8.2% of the phenotypic variation (Table 1, Fig 3). The fifth QTL on the long arm of chromosome 4B was flanked by SNPs *GBS2348* and *GBS3434*, which was significant on spring 2012 only and explained 5.6% of the phenotypic variation (Table 1, Fig 3).

### KASP markers development

**Fig 4:**
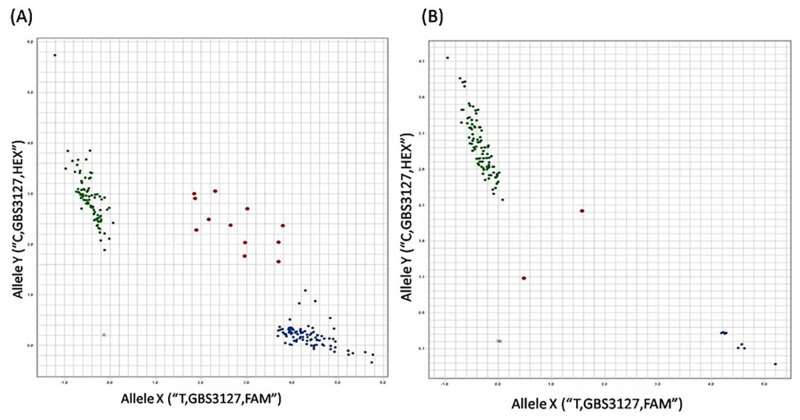
A KASP assay profile of SNP *GBS3127*. A) SNP *GBS3127* in 186 RILs of HYZ x Wheaton; B) SNP *GBS3127* in 96 U.S. wheat association mapping (AM). Blue dots represent T (resistance) allele, green dots represent C (susceptible) allele of *GBS3127*, red dots refer to heterozygotes, and the black crosses are ddH_2_O control.

To verify the accuracy of GBS-SNP data, and fill up the missing data from the GBS-SNPs in the QTL regions, 21 KASP assays were designed according to the corresponding GBS sequences harboring the SNPs that were mapped in the QTL regions on 5AS, 6BS, or 2B-2, and 14 of them segregated among the RILs (Fig 4A). Ten KASP-SNPs (four each in the QTLs 5A and 6B regions, and two in the QTL 2B-2 region) (S2 Table) had identical allele calls with the corresponding GBS-SNPs across the RILs, and four other were mapped outside the QTL regions with five mismatches in *GBS5920* and *GBS2732*, six mismatches in *GBS2577*, and more than ten mismatches in *GBS3018*, thus, these four markers were not pursued further.

The ten KASPs (S2 Table) that were remapped to the three significant QTL regions (5A, 6B, and 2B-2) were then validated in an association mapping (AM) population of 96 U.S. elite wheat accessions as well as four Chinese FHB resistant landraces, Huangcandou, Baishanyuehuang, Huangfangzhu and Wangshuibai. All of the ten KASPs were amplified well in the AM population, and the all four FHB resistant landraces amplified the same alleles as in “HYZ”. Two KASPs on 6B QTL (*GBS4963*, and *GBS4116*) and one KASP on 5A QTL (*GBS2573*) separated into almost equal clusters. Another three KASPs on 5A QTL (*GBS3127*, *GBS5669*, and *GBS1852*) and two on 6B QTL (*GBS0158* and *GBS4305*) showed unequal clusters with more lines in Wheaton allele cluster. Among them, SNPs *GBS3127* on 5A (Fig 4B) and *GBS4305* and *GBS0158* on 6B had all Wheaton alleles in the AM population, except one or two with heterozygous genotypes. Two KASPs from 2B QTL (*GBS5855*, and *GBS1713*) showed unequal clusters with more lines in ‘HYZ’ allele cluster.

### Effects of QTLs on FHB type II resistance

**Fig 5:**
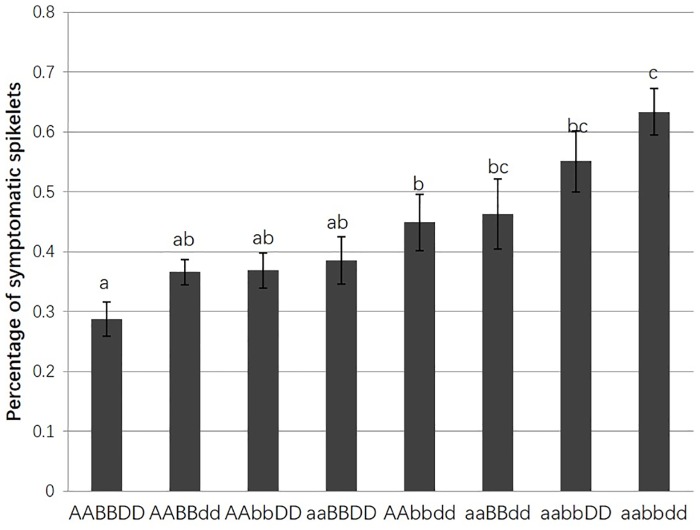
Effects of different combinations of three QTLs on 5A, 6B and 7D for percentage of symptomatic spikelets in a spike (PSS) analyzed in the RIL population. HYZ alleles were assigned as AA (5A), BB (6B) and DD (7D) and ‘Wheaton’ alleles aa (5A), bb (6B) and dd (7D). The solid bars stand for mean PSS of each group, the length of each line refers to standard errors, and different letters indicated significant difference between two genotypes.

To investigate the effect of individual QTLs on FHB resistance, RILs were grouped according to their allele combinations at three repeatable QTLs (5A, 6B, and 7D), and their allele substitution effects were compared among the groups. Eight possible allelic combinations at the three QTLs are designated, AABBDD, AABBdd, AAbbDD, aaBBDD, AAbbdd, aaBBdd, aabbDD and aabbdd; where AA, BB and DD represent ‘HYZ’ alleles at QTLs on 5A, 6B and 7D, respectively (Fig 5). The average PSSs for the eight genotypic groups of RILs ranged from 28.7% to 63.4%. The closet KASP markers to each of the three QTLs were *GBS3127* on 5A, *GBS4305* on 6B and *Xcfd46* on 7D, thus the three markers were used to represent the three QTLs to estimate their allelic effects. The mean PSSs for the genotypic groups that had only one of the three resistance QTLs were 44.9% for 5A, 46.3% for 6B, and 55.1% for 7D (Fig 5); whereas the PSS for the group of RILs with none of the three resistance alleles (“null” group) was 63.4%, suggesting all the three QTLs reduced the FHB severity with the 5A QTL showing the largest effect on FHB resistance.

## Discussion

### Fhb1 is absent in HYZ

Many Chinese wheat cultivars and landraces show a high level of type II FHB resistance, and most of them carry *Fhb1* on the short arm of chromosome 3B [17, 18, 22, 28, 29]. HYZ showed a similar level of resistance as these landraces [17], but *Fhb1* was not mapped in HYZ as in other landraces although the *Fhb1* diagnostic marker (*Umn10*) and flanking markers are polymorphic, which agrees with Li et al. (2011). In the current study, we used a completely new population of larger size (172 vs. 136 RILs) from the same parents as used by Li et al. (2011) and constructed a high-density SSR and GBS-SNP map to remap possible QTLs in HYZ. The results from the current study suggest that a high level of resistance in HYZ is not due to *Fhb1*, a QTL with a major effect on FHB resistance in most Chinese FHB resistant landraces, but due to additive effects of multiple minor QTLs.

### QTLs for type II FHB resistance in HYZ

Among the eight QTLs identified in the current study, the QTL on 5AS explained the largest phenotypic variation (6.1~16.0%) across all three experiments. To date, more than 14 QTLs for FHB resistance have been reported in chromosme 5A and explained 4.5~32% of the phenotypic variation in different experiments [15, 18, 22, 29–34]. Some of them showed type I resistance, but other showed types II or III resistance (low DON content) in different cultivars. Meta-analysis found at least three different QTL clusters [8] with two on the chromosome 5AL as mapped in ‘Renan’ [35] and one mapped near the centromere of 5AS from various sources [18, 29, 33, 36]. The 5AS QTL was further fine mapped in Wangshuibai to a 0.3 cM region flanked by *Xgwm293* and *Xgwm415*, designated as *Fhb5* [15]. In the current study, the QTL on 5A was mapped into a 1.9 cM interval between SNP *GBS3127* and SSR marker *Xbarc316*, however, *Xgwm293* was one of several SSR marker mapped under the peak of the QTL, suggesting the QTL on 5A is most likely *Fhb5*. Besides, three of the four KASPs (*GBS3127*, *GBS5669* and *GBS1852*) tightly linked to 5A QTL amplified susceptible “Wheaton” alleles in almost entire AM population, except in Wangshuibai where the “HYZ” alleles were amplified, indicating these markers are good for marker-assisted selection of *Fhb5* in breeding.

The QTL on 6BS in the current study was assigned to the interval between *GBS4963* and *GBS3704*, which explained 8.3~11.1% of the phenotypic variation in two of the three experiments and the mean PSS. This QTL was mapped very close (~2 cM) to *Xgwm88* and *Xwmc397*, which is linked to *Fhb2*, thus this QTL is most likely *Fhb2* [37]. *Fhb2* has been reported with varied effects ranging from 4.4~24.0% on type II resistance [18, 34, 37–40]. This QTL was previously mapped in a 6.0 cM interval in HYZ [18], however, it is narrowed to a 2.4 cM interval in this study owing to increased marker-density.

The QTL mapped on 7DL in the current population coincides with the major QTL reported by Li et al. (2011), but with a much smaller effect (5.6~7.5%). QTLs on 7D with a minor effect have also been reported for type III resistance in Arina [41] and type IV resistance in Wangshuibai [42], and they are most likely the same QTL as in HYZ in this study because they share the common marker *Xcfd46* [8, 41]. The discrepancy in QTL effect between the current study and Li et al. (2011) might be partially due to the differences in population size and environment conditions for phenotyping. Increase in population size may reduce the effect of a QTL, however, larger population size and higher marker density can improve the estimation accuracy of a QTL effect. Thus, the 7D QTL is most likely a minor QTL for FHB resistance.

Several QTLs have been previously reported on 2B of different populations. One QTL for type II resistance was mapped close to *Xgwm120* on 2BL of Ning7840 [43] and ‘Ernie’ [44], and another QTL was mapped close to *Xgwm210* on 2BS in ‘Renan’ for type II resistance [35] and in ‘Patterson’ x ‘Goldfield’ population for type I resistance [45]. In the current study, two minor QTLs were mapped on the chromosome arm 2BS (QTLs 2B-1 and 2B-2) and they are far from *Xgwm210* and *Xgwm120*, therefore, they were more likely novel QTLs for type II resistance. Interestingly, they are all from the susceptible parent ‘Wheaton’, suggesting that some susceptible cultivars may also harbor minor QTLs for FHB resistance.

The 4D QTL was significant in fall 2012 only. A few QTLs have been reported on chromosome 4D of DH181 for FHB type I and IV resistance [34], in Chinese Spring x SM3-7ADS, Spark’ and ‘Arina’ for FHB type II resistance [41, 46, 47]. However, their allelic relationship remains to be determined because common markers are not available among these QTLs.

The QTL on 3BL closely linked to *Xbarc164* was significant in fall 2012 only. *Fhb1* and a QTL near the centromere have been mapped on 3BS in many studies [11, 22, 28, 29, 34, 48], but only one QTL linked to *Xgwm247* has been mapped on 3BL of Huapei 57–2 [49]. However, *Xbarc164* was far from *Xgwm247* (~100 cM) in 3B reference map (http://wheat.pw.usda.gov/GG3/), therefore, they are not the same QTL and the one identified in the current study is most likely a new QTL.

The QTL tightly linked to *Xgwm6* on 4B showed a minor effect in the spring 2012 experiment. This QTL is more likely *Fhb4* that was previously mapped in ‘Ernie’ [44], ‘Chokwang’ [50], ‘Wangshuibai’ [39, 51], and ‘Wuhan1’ [52] because *Xgwm6* is closely linked to the *Fhb4*-linked marker *Xgwm149* on 4BL5-0.86–1.00 bin [14].

### Conversion of GBS-SNPs into KASP assays

GBS facilitates quick identification of SNPs for QTL mapping and many other applications at a low cost by multiplexing samples using barcodes [16, 53, 54]. However, GBS also generates a large number of missing data across a mapping population due to the limited sequencing depth [16, 55, 56]. The missing data can be predicted through imputation based on available reference genome sequences [56]. However, the wheat reference genome sequences are not complete, and imputed data may not be accurate for QTL mapping. Another way is to increase the number of runs for each library to reduce the number of missing data. In the current study, four Ion Proton runs of this population significantly increased numbers of SNPs when compared with a single run. For a small set of GBS-SNPs that were mapped in the QTL regions, missing data were filled by KASP data that not only eliminated missing data, but also verified the accuracy of GBS-SNP data by comparing the GBS-SNPs with KASP data in the segregating population. Among the 21 KASP assays designed, 14 (67%) were amplified. Seven failed KASP assays are due to that the SNP positions are too close to one end of the sequence reads that cause difficulties in primer design. Ten of the 14 amplified KASPs were remapped to the same positions corresponding to GBS-SNPs mapped, while the other four were not due to either GBS sequencing errors or SNP calling errors. The ten KASPs were then evaluated for their allele distribution in an association mapping (AM) population of 96 U.S. elite lines and cultivars, and four Chinese FHB resistant landraces (Huangcandou, Baishanyuehuang, Huangfangzhu and Wangshuibai). The seven KASP assays separated the Am population into two unequal clusters of HYZ and Wheaton alleles. Five KASP assays (*GBS3127*, *GBS5669* and *GBS1852* for 5A, *GBS0158* and *GBS4305* for 6B) amplified ‘Wheaton’ alleles in most of U.S. elite wheat lines, with only a few or none of the lines amplifying HYZ alleles in the AM population, indicating most of the elite lines/varieties may not have these two QTLs yet and thus, these KASPs can be effectively used to transfer these QTLs into US winter wheat. KASPs *GBS1713* and *GBS5855* linked to 2B-2 amplified HYZ alleles in more lines than the ‘Wheaton’ alleles. Because 2B-2 QTL was contributed by ‘Wheaton’, the HYZ alleles on these two markers were prevalent in the AM population, therefore, these SNPs are good markers for transferring the QTL into U.S. winter wheat. However, an almost equal number of lines amplified each allele of three KASPs (*GBS4963*, *GBS2573*, and *GBS4116*), indicating that these markers are not as informative as previously described ones, but they can still be useful for MAS if the breeding parents are polymorphic.

## Conclusion

HYZ is a highly FHB-resistant Chinese landrace, but *Fhb1* was not mapped in HYZ. Besides three previously mapped QTLs, *Fhb2*, *Fhb4* and *Fhb5*, [18, 39], four putative new minor QTLs were identified on the chromosomes 2B (2), 3B, and 4D in the current study. The results demonstrated that a high level of FHB resistance in HYZ is controlled by multiple minor QTLs with additive effects, thus pyramiding enough minor QTLs can achieve a high level of resistance to FHB. Many adapted cultivars are moderately susceptible and they may contain different minor QTLs for FHB resistance. Stacking these minor QTLs from different cultivars may generate highly resistant cultivars, thus identification of QTLs and linked markers from locally adapted cultivars may provide useful sources of resistance QTLs for breeding. In this study, GBS-SNPs linked to several QTLs have been successfully converted to KASP assays, and they are ready to be used in MAS to pyramid these minor QTLs in breeding programs.

## Supporting Information

S1 Table List of KASP assays developed from GBS-SNP sequences. (XLSX)

S2 Table List of 96 U.S. elite wheat accessions from an association mapping (AM) population. (XLSX)
